# Association between night shift work and markers of metabolism, cardiovascular and immune system in a population-based German cohort

**DOI:** 10.1007/s11357-025-01596-8

**Published:** 2025-03-27

**Authors:** Nora Bittner, Horst-Werner Korf, Susanne Moebus, Börge Schmidt, Svenja Caspers

**Affiliations:** 1 Institute for Anatomy I, Medical Faculty & University Hospital Duesseldorf, Heinrich-Heine-University, Universitaetsstraße 1, 40225 Duesseldorf, Germany; 2 Institute of Neuroscience and Medicine (INM-1), Research Centre Juelich, 52428 Juelich, Germany; 3 Institute of Urban Public Health, University of Duisburg-Essen, 45122 Essen, Germany; 4 Institute for Medical Informatics, Biometry and Epidemiology, University Hospital Essen, University of Duisburg-Essen, 45130 Essen, Germany

**Keywords:** Anthropometric and blood values, Interaction with sex, Night shift work, Risk for human health

## Abstract

In humans, night shift work is a major reason for chronodisruption, may affect health and increase the risk of a metabolic syndrome, but results obtained so far are ambiguous. In this population-based, cross-sectional study, PRESENT and FORMER shift workers were compared to age- and sex-matched controls, who never worked in shift with regard to body mass index, waist-hip-ratio total, high-density lipoprotein and low-density lipoprotein, cholesterol and C-reactive protein. Moreover, association with sex, length of shift work and medication were investigated. The present results do not support the hypothesis that night shift work per se is associated to an increased risk of metabolic syndrome, and cardiovascular and immune malfunctions: no differences were found in mean anthropomteric and blood values between present or former shift workers and respective matched controls. When analyzing the proportion of participants showing values beyond the clinically relevant cut-offs, no general effect of shift work was observed, but the data may suggest an interaction between shift work and sex. These divergent results may be due to differences in the socio-economic status, the health care system and the shift schedule. All these parameters need to be considered in future studies addressing the impact of night shiftwork on human health.

**Supplementary Information:**

The online version contains supplementary material available at 10.1007/s11357-025-01596-8.

## Introduction

In humans, shift work is a major reason for chronodisruption, also called circadian misalignment and considered as a risk factor for metabolic, cardiovascular and immune malfunctions [11], [12]; [14, 16, 19, 23–25, 29, 30, 34]. Subclinical abnormalities in HbA1c and changes in expression of circadian clock genes were reported in current night shift workers (NSWs) as compared to former NSWs and individuals who work during daylight hours only [20]. A strong relationship between the circadian system and metabolism has also been demonstrated in animal models. Genetic disruption of the circadian clock predisposes rodents to metabolic disease [21, 27] and exposure to artificial light at night promoted significant metabolic disturbances [10, 17].

However, some aspects about the association between night shift work and metabolic malfunctions remain open. Thus, the sex of the shift workers may play an important role and most studies addressing the association between night shift work and metabolic, cardiovascular and immune malfunctions were performed with females (nurses). Interestingly, a study on male railway workers failed to show that long-term night shift work is associated with a significantly increased risk of the metabolic syndrome [6]. Moreover, retrospective studies comparing a large cohort of male night shift workers with non-shift workers in a German chemical company did not provide evidence for a carcinogenic effect of night shift [32], an excessive risk of mortality from cancer [32] and non-cancer diseases, especially ischemic heart disease [15, 33] in night shift workers.

In this study, we have evaluated the association between night shift work, metabolism, and the cardiovascular and immune systems in the population-based Heinz-Nixdorf Recall study (HNR; [22]) and the related Heinz-Nixdorf-recall Multigeneration study (HNR-MGS). Participants working in night shift were compared with age- and sex-matched controls who never worked in night shift. Data recorded were as follows: systolic and diastolic blood pressure, body mass index (BMI), waist-hip ratio, levels of HbA1c, fasting blood glucose, total HDL and LDL cholesterol, LDL/HDL ratio, levels of triglycerides, uric acid, C-reactive protein and number of erythrocytes and white blood cells. To investigate sex differences, we calculated interaction effects between sex and shift work group and investigated the relationship between all parameters and the length of shift work. Finally, we addressed the question whether shift workers needed more drugs targeting metabolic, cardiovascular, metabolic and immune functions than subjects who were never engaged in shift work.

## Methods

### Participant selection

The data of this study were collected from the population-based HNR study (total number of participants at the second follow-up examination: 3087, males:1507, females: 1580), as well as the related HNR-MGS study (total number of participants at baseline examination, 2897; males, 1306; females, 1591). The study protocol of the Heinz-Nixdorf Recall and Heinz-Nixdorf-recall Multigeneration studies was approved by the Ethics Committee of the University of Duisburg-Essen, Germany, and is in accordance with the declaration of Helsinki. Written informed consent was obtained from all participants prior to inclusion in the study.

The current investigation enrolled subjects from both cohorts who worked in night shift and age- and sex-matched controls who never worked in night shift and for whom the following data were available: systolic and diastolic blood pressure, BMI, waist-hip ratio, HbA1c, levels of fasting blood glucose, total HDL and LDL cholesterol, LDL/HDL ratio, triglycerides, uric acid, C-reactive protein as well as number of erythrocytes and white blood cells and percentage of granulocytes.

Shift work parameters were obtained in an interview in which the participants were asked whether they worked in shift at any time of their life (“Yes”/ “Never”), with shift being defined as a work schedule outside the period between 7am and 6 pm. Participants who answered “Yes” were asked (i) which shift schedule they were engaged in (rotating shifts without night shifts, rotating shifts including night shifts or early shifts, late shifts and night shifts only), (ii) how many years they worked in shift and (iii) whether they worked in shift at time of data acquisition. The present study includes participants who worked either in night shifts only or in rotating shifts including night shifts, since night shifts are the greatest challenge for the human circadian system and therefore have the greatest impact on health parameters [8]. Details on numbers of missing values for each variable of interest are given in a flow chart (Suppl. Figure 1).

This resulted in three groups (Table 1): (1) control group including participants, who had never been engaged in shift work (NEVER shift workers, overall *n* = 3266; males, 1386; females, 1880); (2) participants who worked in night shift at the time of data acquisition or within the last year before (PRESENT shift workers, *n* = 125; males, 77; females, 48); (3) participants who had stopped working in night shifts 2 or more years before the time point of the data acquisition (FORMER shift workers, *n* = 662; males, 493; females, 169). To compare shift workers and non-shift workers, we employed a propensity score matching between both groups using the “matchit”-algorithm in R statistics. To obtain matched controls for PRESENT shift workers, we submitted the age and sex data for the available 125 PRESENT shift workers and for the elegible 3406 NEVER shift workers. The algorithm selected 125 participants as matched controls for PRESENT shift workers, here termed “matched NEVER_Pres_.” The same was done for FORMER shift workers: Here age and sex data for FORMER shift workers as well as for participants, who never worked in shift were submitted to matchit, resulting in *n* = 662 matched controls for the FORMER shift workers, here termed “matched NEVER_FORM_” (Table 1). Hence, in total, 787 NEVER shift workers were selected as matched controls for PRESENT and / or for FORMER shift workers.

**Table 1:** Number and age of male and female shift workers and matched controls of the total sample, as well as only participants without relevant medication. Age is given in mean years with standard deviation in brackets

	PRESENT	Matched NEVER_PRES_	FORMER	Matched NEVER_FORM_
Total sample	125	125	662	662
*n* males/females	77/48	77/48	493/169	493/169
Age	46.7 (12.06)	46.7 (12.06)	61.30 (13.48)	61.30 (13.48)
Age range	22–71	22–71	20–86	20–85
Participants without medication				
Sample size	69	69	212	212
*n* males/females	46/23	46/23	152/60	152/60
Age	42.6 (10.53)	42.3 (11.21)	53.26 (13.10)	54.60 (13.90)
Age range	22–68	23–68	25–84	26–82

Systolic and diastolic blood pressure were recorded using an OMRON® system. Body mass index (BMI) and waist hip ratio were calculated from of weight, height, hip and waist circumference. Levels of HbA1c (%), fasting blood glucose (mg/dl), total HDL and LDL cholesterol (mg /dl), LDL/HDL ratio, triglycerides (mg/dl), uric acid (mg/dl) and C-reactive protein (mg/dl) and number of erythrocytes and white blood cells (#/nl) and percentage of granulocytes (%) were measured in blood samples taken in the morning.

Further, information on the use of drugs targeting metabolism, cardiovascular and immune functions was collected. These drugs include antihypertensive drugs, diuretics, statins, xanthine oxidase inhibitors, oral antidiabetics, insulin and acetylsalicyl acid (ASS). They are denominated here as “relevant medications.” Numbers, age and sex of participants who did not use any of these drugs are given for each group in Table 1.

### Statistical analysis

#### Analyses of matched samples

1. We compared PRESENT (*n* = 69) to matched NEVER_PRES_ shift workers and FORMER (*n* = 212) to matched NEVER_FORM_ shift workers, who did not use any relevant medications by use of two multivariate analyses of covariance (MANCOVA). Sex and shift work group were used as between-subject factors, while age was introduced as covariate. Systolic and diastolic blood pressure, body mass index (BMI), waist-hip ratio, levels of HbA1c (%), fasting blood glucose (mg/dl), total HDL and LDL cholesterol (mg/dl), LDL/HDL ratio, triglycerides (mg/dl), uric acid (mg/dl) and C-reactive protein (mg/dl) and number of erythrocytes and white blood cells (#/ nl) and percentage of granulocytes (%) were introduced as dependent variables. To investigate sex differences, we calculated interaction effects by introducing an interaction term between shift work group and sex into the MANCOVA model.
2. We analyzed the number of participants without relevant medication whose values in anthropometric and blood values were different from the critical cutoffs (Table 2) defined according to recent literature and suggestions by the World Health Organization (WHO) [31]. Particular emphasis was paid to the metabolic syndrome defined by the presence of central obesity, elevated systolic or diastolic blood pressure, elevated levels of triglycerides or fasting glucose or decreased HDL cholesterol levels [1]. Chi-square (*χ*^2^) tests were used to test for differences between the groups (PRESENT vs. matched NEVER_PRES_; FORMER vs. matched NEVER_FORM_ shift workers) in proportions of participants showing values different from the critical cutoffs. This evaluation was repeated after stratifying for sex.
3. We investigated the relationship between anthropometric and blood values and the length of shift work in participants without relevant medication. Length of shift work was defined by the self-reported number of years, which the participants worked in shift (number of shift work years). We defined two groups of shift work length with (1) high number of shift work years versus (2) low number of shift work years for PRESENT and FORMER shift workers independently by calculating the mean number of shiftwork years. However, using one cutoff for all PRESENT shift workers resulted in much less females (*n* = 7) as compared to males (*n* = 20) in the “high shift work length” group, since males worked in shift work for longer time than females. The same was true for FORMER shift workers (“high shift work length”: *n* = 16 females, *n* = 49 males). Thus, we calculated sex-specific means of shift work length for PRESENT (males = 16.1 years; females = 10.7 years) and for FORMER (males = 8.2 years; females = 6.5 years) shift workers. Long versus short shift work length was then used as independent variable, age and sex as covariates and each anthropometric and blood value as dependent variable for PRESENT and FORMER shift workers without relevant medication. Additionally, we calculated the interaction between shift work length groups and sex.
4. We addressed the question whether shift workers used more drugs than NEVER shift workers.
5. Sensitivity analysis in shift workers with and without medication use. As a sensitivity analysis, we repeated the analysis of mean differences as well as the association with shift work years for all available shift workers and their matched controls (with and without relevant medication, *n* = 125 for PRESENT and NEVER_PRES_;* n* = 662 for FORMER and NEVER_FORM_) and used medication as covariate to control whether we obtained stable results.
6. Analyses of all available participants. We calculated one omnibus MANCOVA model comparing PRESENT and FORMER shift workers directly to all available NEVER shift workers, thus introducing shift work group as a factor with three levels (PRESENT, FORMER, NEVER_ALL_ shift workers), using sex as additional factor and age as covariate.

**Table 2:** Definition of critical cutoff levels for each parameter or syndrome

Parameter/syndrome	Cutoff definition
Central obesity	Waist circumference ≥ 90 cm (males) and ≥ 80 cm (females) OR if BMI was above 30 kg/m
Hypertension	≥ 140/90 mmHg (Joint National Committee, JNC; Chobanian et al., [4])
Obesity	BMI ≥ 25 kg/m square (WHO 2000)
Elevated systolic BP	≥ 130 mmHg
Elevated diastolic BP	≥ 85 mmHg
Elevated triglycerides	> 150 mg/dl
Elevated fasting glucose	≥ 100 mg/dl
Decreased HDL cholesterol	< 40 mg/dl (males) and < 50 mg/dl (females)
Uric acid	> 7 mg/dl

Within this analysis, we estimated the regression parameters for the main effect of the three shift work groups. The regression weights serve as an estimate for the association strength between the shift work status (PRESENT, FORMER, NEVER_ALL_) and health parameters. Further, we introduced the interaction term between sex and shift work group, i.e., for each combination of their levels (2 × 3). The concept of this analysis was to introduce all covariates and factors into one overall model using the largest sample size for the non-shift working group to obtain a precise estimation of variability within this group.

In additional supplementary analyses, education [5, 18, 26, 28] was added as a covariate and family status [13] was added as a random effect with a constant term, since the two samples examined here (MGS and HNR) contain participants with family relationships.

## Results

### Participants without medication

These comprised 69 PRESENT shift workers (males, 46; females, 23), 212 FORMER shift workers (males, 152; females, 60) and the respective matched controls (NEVER_PRES_ and NEVER_FORM_) (Table 1).

#### PRESENT versus matched NEVER shift workers

None of the mean values of the anthropometric and blood parameters differed significantly between PRESENT and NEVER_PRES_ workers (MANCOVA, all values* p* > 0.098, Table 3).

**Table 3:** Mean values for each parameter, as well as *p*-values for the group differences between PRESENT and matched NEVER_PRES_ shift workers (corrected for age and sex), as well as the interaction term between sex and shift work group (PRESENT versus NEVER_PRES_ (corrected for age) as obtained with MANCOVA with Bonferroni correction for multiple comparisons, *p < 0.05

	Mean values		*p*-value	
Parameter	PRESENT	NEVER_PRES_	Group differences	Interaction term
Systolic BP [mmHg]	121.41	119.20	0.201	0.717
Diastolic BP [mmHg]	73.05	73.44	0.775	0.494
LDL/HDL ratio	2.13	2.21	0.609	0.178
Body mass index [kg/m^2^]	26.16	25.68	0.504	0.940
Waist-hip-ratio	0.85	0.85	0.556	0.630
HDL cholesterol [mg/dl]	60.77	57.35	0.137	0.040*
LDL cholesterol [mg/dl]	121.16	118.68	0.661	0.878
Total cholesterol [mg/dl]	196.13	192.51	0.529	0.572
Triglycerides [mg/dl]	94.55	104.89	0.402	0.763
HbA1C [%]	5.27	5.28	0.582	0.741
Glucose (serum) [mg/dl]	91.74	93.17	0.961	0.667
Uric acid [mg/dl]	5.36	5.33	0.468	0.272
C-reactive protein [mg/dl]	0.12	0.23	0.098	0.283
Leukocytes [#/ nl]	4.68	4.75	0.291	0.223
Erythrocytes [#/ nl]	5.81	5.49	0.152	0.244
Basophilic granulocytes [%]	0.58	0.53	0.413	0.623
Eosinophilic granulocytes [%]	2.79	2.96	0.659	0.247
Neutrophilic granulocytes [%]	56.71	56.25	0.737	0.692

**Fig. 1:**
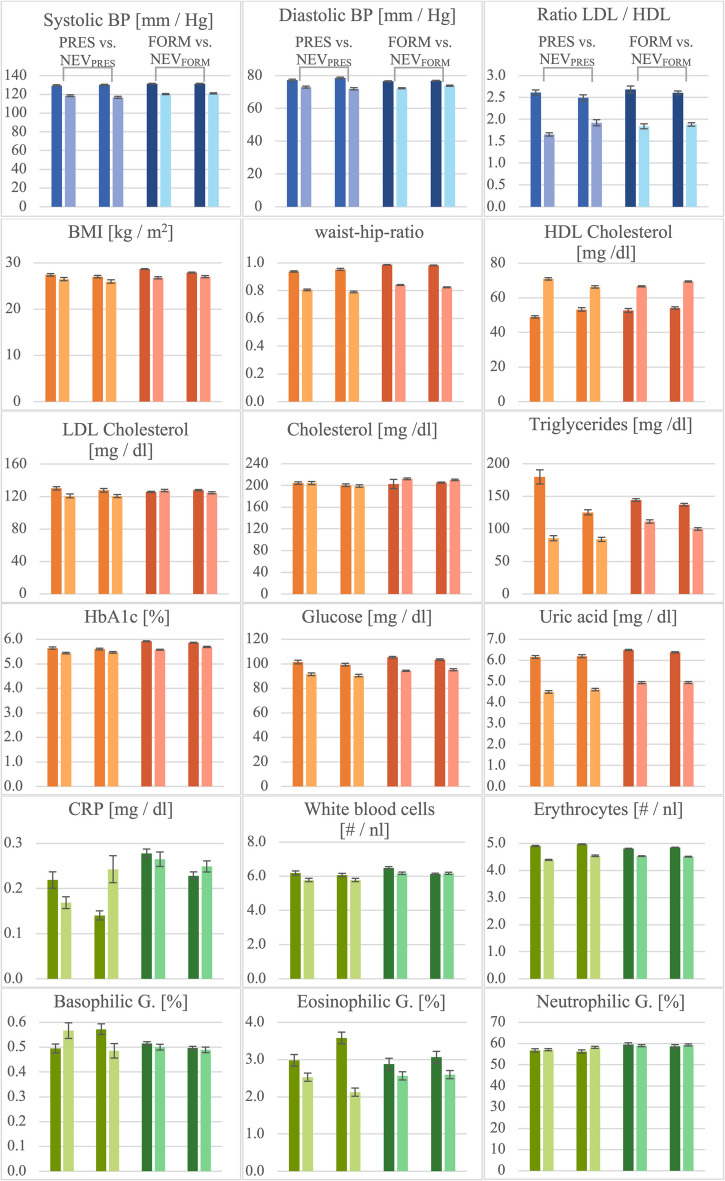
Mean values of cardiovascular (orange), metabolic (blue) and immune system parameters (green) in PRESENT versus matched NEVER_PRES_ and FORMER versus matched NEVER_FORM_ shift workers. Error bars represent standard errors of the mean. Male participants are depicted in darker and female participants in lighter colors

Between subjects-effects indicated a significant interaction effect between shift work group and sex in HDL (*p* = 0.040): male participants showed lower HDL values than female participants. Levels of HDL were comparable between male PRESENT and matched NEVER_PRES_ shift workers. In contrast, female PRESENT shift workers showed lower HDL values than female NEVER_PRES_ shift workers. No significant interaction effects were found for all remaining anthropometric and blood values (Fig. 1).

#### FORMER versus matched NEVER shift workers

None of the mean values of the anthropometric and blood parameters differed significantly between FORMER to matched NEVER_FORM_ shift workers (MANCOVA, all values *p* > 0.124, Table 4, Fig. 1).

**Table 4:** Mean values for each parameter, as well as *p*-values for the group differences between FORMER and matched NEVER shift workers (corrected for age and sex), as well as the interaction term between sex and shift work group (FORMER versus NEVER covariance (corrected for age) as obtained with multivariate analysis with Bonferroni correction for multiple comparisons. *p < 0.05

	Mean values		*p*-value	
Parameter	FORMER	NEVER_FORM_	Group differences	Interaction term
Systolic BP [mmHg]	126.13	126.53	0.492	0.625
Diastolic BP [mmHg]	74.32	75.23	0.395	0.517
LDL/HDL ratio	2.24	2.26	0.910	0.688
Body mass index [kg/m^2^]	27.87	27.62	0.889	0.039*
Waist-hip ratio	0.92	0.91	0.314	0.963
HDL cholesterol [mg/dl]	59.84	61.93	0.740	0.658
LDL cholesterol [mg/dl]	126.61	126.31	0.127	0.519
Total cholesterol [mg/dl]	207.94	208.27	0.124	0.441
Triglycerides [mg/dl]	128.31	119.00	0.591	0.762
HbA1C [%]	5.78	5.81	0.425	0.157
Glucose (serum) [mg/dl]	100.27	99.75	0.129	0.564
Uric acid [mg/dl]	5.74	5.68	0.659	0.610
C-reactive protein [mg/dl]	0.28	0.25	0.212	0.392
Leukocytes [#/nl]	6.33	6.16	0.914	0.055
Erythrocytes [#/nl]	4.66	4.67	0.492	0.924
Basophilic granulocytes [%]	0.51	0.49	0.875	0.361
Eosinophilic granulocytes [%]	2.72	2.83	0.864	0.264
Neutrophilic granulocytes [%]	59.49	59.22	0.271	0.068

Between subjects’ effects indicated a significant interaction effect between shift group and sex only for BMI (*p* = 0.039, Table 4). Notably, female FORMER shift workers showed the lowest BMI as compared to female NEVER_FORM_ or male FORMER or NEVER_FORM_ shift workers (Fig. 1). There were no significant interaction effects for all other anthropometric or blood parameters in any setup.

#### PRESENT versus FORMER versus matched NEVER shift workers

When comparing PRESENT, FORMER and all matched NEVER shift workers directly using an omnibus MANCOVA test, the interaction effect between shift work group and sex in HDL levels was no longer significant; all other results did not change.

### Participants with values different from the critical cutoff

The prevalence for the defined critical cutoffs was comparable between PRESENT/matched NEVER and FORMER/matched NEVER shift workers (Table 5a, b). The additional chi-square (*χ*^2^) test hinted at a significantly higher proportion of HDL values above cutoff for NEVER than for PRESENT shift workers (*p* = 0.028). This was mainly driven by five female NEVER shift workers showing HDL above cutoff compared to zero female PRESENT shift workers (*p* = 0.018). For FORMER shift workers, a significantly higher proportion of male FORMER shift workers showed values above cutoff for waist circumference than NEVER shift workers (*p* = 0.032). All other parameters, including the prevalence of metabolic syndrome did not differ significantly.

**Table 5:** Number of participants whose values were different from the critical cutoffs, (a) in PRESENT shift workers and matched NEVER_PRES_ shift workers, (b) in FORMER shift workers and matched NEVER_FORM_ shift workers. *f* female, *m* males; *significant below *p* = 0.05 in chi-square test

a					
Parameter	# PRESENT	# NEVER_PRES_	*p* (*χ*^2^)	*p* (*χ*^2^) m	*p* (*χ*^2^) f
Systolic BP ≥ 130 mmHg	20; m = 18; f = 2	16; m = 15; f = 1	0.337	0.388	0.550
Diastolic BP ≥ 85 mmHg	9; m = 9; f = 0	4; m = 4; f = 0	0.145	0.135	No cases
BMI > 25	39; m = 28; f = 11	32; m = 22; f = 10			
BMI > 30	12; m = 6; f = 6	13; m = 8; f = 5	0.825	0.562	0.730
Waist circumference ≥ 90 cm (m); ≥ 80 cm (f)	37; m = 24; f = 13	37; m = 24; f = 13	0.999	0.999	0.99
HDL cholesterol < 40 mg/dl (m); < 50 mg/dl (f)	2; m = 2; f = 0	9; m = 4; f = 5	0.028	0.398	0.018*
Total cholesterol. > 200 mg/dl	34; m = 26; f = 8	26, m = 21; f = 5	0.170	0.297	0.326
Triglycerides > 150 mg/dl	11; m = 11; f = 0	16; m = 14; f = 2	0.283	0.482	0.148
HbA1C; 6.5%	0; m = 0; f = 0	1; m = 1; f = 0	0.316	0.315	No cases
Glucose (serum) ≥ 100 mg/dl	14; m = 11; f = 3	11; m = 8; f = 3	0.507	0.440	0.999
Uric acid > 7 mg/dl	12; m = 12; f = 0	8; m = 8; f = 0	0.333	0.312	No cases
Metabolic Syndrome	12; m = 11; f = 1	9; m = 9; f = 0	0.73	0.694	0.999
b					
Parameter	# FORMER	# NEVER	*p* (*χ*^2^)	*p* (*χ*^2^) m	*p* (*χ*^2^) f
Systolic BP ≥ 130 mmHg	82; m = 70; f = 12	83; m = 69; f = 14	0.921	0.909	0.658
Diastolic BP ≥ 85 mmHg	38; m = 35; f = 3	36; m = 30; f = 6	0.798	0.485	0.298
BMI > 25	135; m = 113; f = 22	115; m = 93; f = 22			
BMI > 30	41; m = 34; f = 7	37; m = 25; f = 12	0.616	0.192	0.211
Waist circumference ≥ 90 cm (m); ≥ 80 cm (f)	153; m = 118; f = 35	134; m = 100; f = 34	0.050	0.032*	0.853
HDL cholesterol < 40 mg/dl (m); < 50 mg/dl (f)	27; 17 (m); f = 10	18; m = 14; f = 4	0.156	0.815	0.155
Total cholestrol > 200 mg/dl	124; m = 91; f = 33	133; m = 93; f = 40	0.373	0.075	0.190
Triglycerides > 150 mg/dl	53; m = 48; f = 5	51; m = 42; f = 9	0.822	0.452	0.255
HbA1C; 6.5%	6; m = 5; f = 1	3; m = 2; f = 1	0.312	0.251	0.999
Glucose (serum) ≥ 100 mg/dl	65; m = 51; f = 14	50; m = 40; f = 10	0.058	0.570	0.471
Uric acid > 7 mg/dl	29; m = 29; f = 0	26; m = 26; f = 0	0.665	0.655	No cases
Metabolic Syndrome	47; m = 44; f = 3	44; m = 41; f = 3	0.622	0.716	0.697

### Analyses of shift work length

We then analyzed the association between number of shift work years and metabolism, cardiovascular and immune system parameters in PRESENT and FORMER shift workers without relevant medication by splitting the respective shift workers into a group with short versus long shift work length.

Within the group of PRESENT shift workers, the overall model was not significant and we did neither find any main effect of shift work length, nor any interaction effect with sex (Table 6).

**Table 6:** Comparison of mean parameter values between PRESENT shift workers with (1) short versus (2) long shift work length. Mean values for each parameter, as well as p-values for the group differences (corrected for age and sex), as well as the interaction term between sex and shift work group (corrected for age) as obtained with multivariate analysis with Bonferroni correction for multiple comparisons. *p < 0.05

	Mean values		*p*-value	
Parameter	Low length	High length	Group differences	Interaction term
Systolic BP [mmHg]	119.348	124.394	0.098	0.618
Diastolic BP [mmHg]	71.843	74.564	0.257	0.066
LDL/HDL ratio	2.049	2.251	0.373	0.977
Body mass index [kg/m^2^]	25.702	26.763	0.398	0.468
Waist-hip ratio	0.846	0.847	0.958	0.503
HDL cholesterol [mg/dl]	62.071	59.248	0.489	0.140
LDL cholesterol [mg/dl]	118.529	125.380	0.462	0.185
Total cholesterol [mg/dl]	192.811	201.679	0.354	0.055
Triglycerides [mg/dl]	84.677	107.268	0.233	0.110
HbA1C [%]	5.326	5.416	0.314	0.592
Glucose (serum) [mg/dl]	89.990	94.156	0.091	0.697
Uric acid [mg/dl]	5.056	5.562	0.141	0.430
C-reactive protein [mg/dl]	0.119	0.128	0.852	0.638
Leukocytes [#/nl]	5.482	6.264	0.166	0.620
Erythrocytes [#/nl]	4.658	4.701	0.641	0.741
Basophilic granulocytes [%]	0.616	0.533	0.524	0.199
Eosinophilic granulocytes [%]	2.834	2.750	0.911	0.731
Neutrophilic granulocytes [%]	3.063	3.713	0.098	0.563

Within FORMER shift workers, the overall model as well as between subject effect did not indicate any significant differences between short compared to long shift work length. However, between subject effects indicated a significant interaction with sex (Table 7); i.e., levels of erythrocytes were lower for males with long compared to short shift work length, while for females levels of erythrocytes were higher in the group with long shift work length (*p* = 0.007). However, all mean levels as well as standard deviations for male and female FORMER shift workers were in the normal range.

**Table 7:** Comparison of mean parameter values between FORMER shift workers with (1) short versus (2) long shift work length. Mean values for each parameter, as well as *p*-values for the group differences (corrected for age and sex), as well as the interaction term between sex and shift work group (corrected for age) as obtained with multivariate analysis with Bonferroni correction for multiple comparisons. *p < 0.05

	Mean values		*p*-value	
Parameter	Low length	High length	Group differences	Interaction term
Systolic BP [mmHg]	124.93	123.46	0.563	0.777
Diastolic BP [mmHg]	75.33	74.48	0.562	0.344
LDL/HDL ratio	2.25	2.28	0.940	0.676
Body mass index [kg/m^2^]	26.19	25.74	0.539	0.528
Waist-hip ratio	0.88	0.90	0.205	0.134
HDL cholesterol [mg/dl]	63.65	62.97	0.810	0.232
LDL cholesterol [mg/dl]	125.99	129.10	0.642	0.953
Total cholesterol [mg/dl]	209.17	210.13	0.886	0.806
Triglycerides [mg/dl]	109.99	101.36	0.466	0.384
HbA1C [%]	5.51	5.70	0.160	0.403
Glucose (serum) [mg/dl]	95.23	99.11	0.344	0.368
Uric acid [mg/dl]	5.46	5.37	0.623	0.491
C-reactive protein [mg/dl]	0.27	0.17	0.218	0.797
Leukocytes [#/nl]	5.93	6.34	0.531	0.944
Erythrocytes [#/nl]	4.69	4.76	0.283	0.007*
Basophilic granulocytes [%]	0.52	0.48	0.480	0.681
Eosinophilic granulocytes [%]	2.74	2.34	0.233	0.773
Neutrophilic granulocytes [%]	3.41	3.56	0.530	0.086

### Number of medication used

**Fig. 2:**
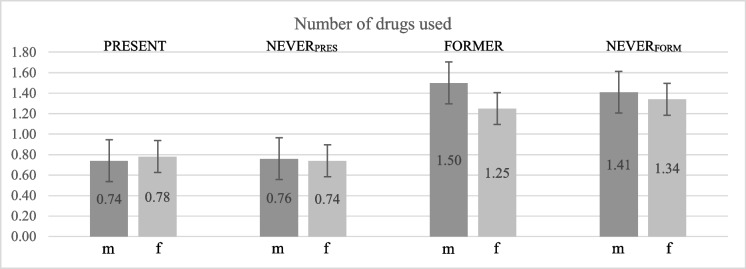
Number of relevant medications used by PRESENT and FORMER shift workers and their respective controls, split up for males (dark grey) and females (grey). Error bars represent standard errors

Finally, we examined whether shift workers took more medication than NEVER shift workers in the whole sample. We therefore used the total number of relevant drugs as dependent variable in the above described ANCOVA model. The number of drugs taken did not differ between PRESENT/matched NEVER shift workers (*p* = 0.955) or FORMER/matched NEVER shift workers (*p* = 0.994). FORMER shift workers and matched controls took more drugs than PRESENT shift workers and matched controls (Fig. 2). We tested whether this was related to the older age of FORMER shift workers and matched controls by means of ANCOVA using shift work group (levels: PRESENT, FORMER, matched NEVER) and sex as factors, and age as covariate. Number of medication was not associated to shift work group as long as age was introduced as a covariate, which was highly significant related to older age (*p* < 0.0001). This association between age and number of medication was not restricted to a specific type of medication, but was generally found for relevant drugs.

### Analyses of PRESENT and FORMER shift workers with and without medication

Within the sensitivity analyses, we repeated our comparison between shift workers and MATCHED controls using all shift workers, i.e., regardless of medication.

Here, the results for PRESENT versus NEVER_PRES_ shift workers were largely the same as for participants without relevant medication, which was also not altered by adjusting for the number or type of medication. However, we found additional interaction effects with sex for LDL-HDL ratio with female PRESENT shift workers presenting the lowest values and levels of CRP (see Suppl. Table 1, Suppl. Figure 2).

For FORMER and NEVER_FORM_ shift workers, the results were also largely the same as for participants without relevant medication, which was also not altered by adjusting for number or type of medication, education and family status (Suppl. Table 2, Suppl. Figure 3).

### Analyses of all available particpants

Comparing all three groups—PRESENT, FORMER and all NEVER shift workers (*n* = 3266)—directly using an omnibus MANCOVA test, correcting for education as covariate and family status as a random effect, resulted in largely the same pattern (Suppl. Table 3) as obtained when comparing PRESENT shift workers with and without medication with NEVER_PRES_. There was an additional hint for sex-specific differences in triglyceride levels within PRESENT shift workers (Suppl. Figure 4).

#### Regression estimates

Estimating regression weights for the association between membership in one shift work group (PRESENT, FORMER, NEVER_ALL_) and the level of the respective health parameter did not reveal additional results.

## Discussion

Night shift work is a major challenge to the circadian system and may affect human health. This topic is important for both individuals and the society. It has thus been addressed in several studies which, however, have revealed controversial results. In the present study, we compared markers for cardiovascular, metabolic and immune functions of night shift workers with age- and sex-matched controls participating in the population-based Heinz-Nixdorf Recall study (HNR) and the related Heinz-Nixdorf-recall Multigeneration study (HNR-MGS) in a cross-sectional cohort design. Particular attention was paid to the metabolic syndrome which refers to the clustering of several known cardiovascular risk factors, including insulin resistance, obesity, dyslipidemia and hypertension. The mean values of all parameters investigated here (systolic and diastolic blood pressure, body mass index, waist hip ratio, levels of HbA1c, blood glucose, total cholesterol, HDL and LDL cholesterol, LDL/HDL ratio, triglycerides, uric acid, C-reactive protein and numbers of erythrocytes and white blood cells and percentage of granulocytes) were not significantly different between night shift workers and age- and sex-matched controls. These results do not support the hypothesis that night shift work is generally associated with an increased risk for metabolic, cardiovascular and immune malfunctions. The mean values of nearly all parameters investigated were in the normal range in both shiftworkers and matched controls indicating similarly good health conditions in all groups.

FORMER shift workers (mean age = 53.26) were significantly older than PRESENT shift workers (mean age = 42.6). Hence, we did not compare PRESENT to FORMER shift workers directly in our main analysis. Slightly elevated values for BMI were observed in PRESENT shift workers and in matched controls. Values for BMI were higher in FORMER shift workers, who also showed increased values for total cholesterol, but again a very similar elevation of these values was also observed in the matched controls. Thus, this increase in BMI and total cholesterol is primarily related to age. When comparing shift work groups (PRESENT and FORMER) directly with selected controls and controlling for age, there was no mean difference in BMI which could be specifically attributed to shift work.

PRESENT shift workers had different proportion of males (66.6% males) than NEVER shift workers (42.2%, before matching) and FORMER shift workers (71.7% males). Since sex had been considered one influencing factor [12], we matched for sex to balance the higher proportion of males in shift versus non-shift workers. To still be able to investigate the differences between male and female shift workers, we analyzed interaction effects within the analyses of covariance. Our data revealed very few interaction effects between sex and shift work. In PRESENT and matched NEVER shift workers, male participants showed generally lower HDL values than female participants, but between male PRESENT and NEVER shift workers HDL levels did not differ. Female PRESENT shift workers showed lower HDL values than female NEVER shift workers within the interaction effect (*p* = 0.040). Thus, HDL levels were significantly different depending on the shift status of women, while this was not true for men. In our sensitivity analyses, the differences in HDL between shift workers and controls turned out to be marginal; i.e. female PRESENT shift workers had a mean HDL level of 60.77 mg/dl, while female NEVER shift workers presented a level of 57.35 mg/dl. Thus, both groups presented HDL values above the clinical cut-off value of 40 mg/dl; i.e. both groups presented mean HDL levels in the normal range. Further, the statistical significance is rather low (*p* = 0.04) since the sample of female shift workers in the current study is smaller (*n* = 23 females) than the male sample. However, the interaction analyses emphazised that the differences between males and females seem to be greater in shift workers than in controls for certain parameters or may even be reversed, e.g. for levels of CRP; hence, both sexes should be investigated on their own in future studies.

For FORMER and matched NEVER shift workers, there was one significant interaction effect between shift work group and sex for BMI (*p* = 0.039): female FORMER shift workers showed the lowest BMI as compared to female NEVER or male FORMER or NEVER shift workers but this interaction effect could no longer be found in our sensitivity analyses. Notably, we found an interaction with sex on levels of HbA1c, where the differences between males and females in shift workers (FORMER) was again greater than in matched controls. Again female FORMER shift workers showed the lowest levels of HbA1c.

Our results may therefore suggest small differences between male and female shift workers, but the statistical significance was rather low as were the effect sizes. When analyzing the proportion of participants with parameter values in the normal range, we found a higher percentage of male shift workers with elevated values of BMI, WHR and glucose as compared to female shift workers and controls. Yet, only few of these proportions were statistically significant: One surprising result was that more female NEVER shift workers showed lower HDL values than female PRESENT shift workers, indicating better health conditions for female PRESENT shift workers. For male participants, only the higher proportion of FORMER shift workers with larger hip circumference was significant, but not the elevated levels in BMI and glucose levels (in line with our sensitivity analyses).

We also examined whether the length of shift work (i.e. short versus long shift work length) would be an influencing factor with regard to general health, since length of shift work may also be an important factor: Some studies have suggested that short periods in night shift work do not increase the risk for cardiovascular disease e.g. [6], [9]. On the contrary, one might argue that a longer employment in shift work may allow the human body to adapt to the work schedule and this adaptation might decrease the risk to develop metabolic syndrome or cardiovascular diseases, as the human body has more time to adopt to the work schedule. The present data did not reveal medically relevant difference associated to shift work length: i.e. we found an interaction effect between length of shift and sex for levels of erythrocytes, but all other levels as well as the differences were in the normal range. Hence, this effect should not be overinterpreted and needs to be further examined in future studies. It again points to the additional insights that can be obtained by considering sex an influencing factor of interest.

Our data from participants who did not take any medications targeting metabolic, cardiovascular and immune functions suggest that night shift work per se is not associated to systematic differences in risk markers for metabolic syndrome, immune or cardiovascular system. This is supported by the finding that night shift workers did not need more drugs than participants who never worked in shift. Further, the results of our sensitivity analyses in all available shift workers and matched controls hinted into the very same direction, even when controlling for medication use as covariate, adding education, family status and obting for the largest sample size.

The present results are in line with retrospective studies comparing a large cohort of male night shift workers with non-shift workers in a German chemical company. These did not provide evidence for a carcinogenic effect of night shift [32] and reported no excessive risk of mortality from cancer [32] and non-cancer diseases, especially ischemic heart disease [15, 33] in night shift workers. Moreover, our previous study investigating a subsample of the cohort used in the current investigation did not show a general association between night shift work and brain function either [3].

However, some studies report that shift work has a negative impact on human health due to several biological and environmental changes. A systematic review of 12 studies evaluated the cross-sectional association between shift work and the prevalence of metabolic syndrome between day and shift workers, specifically employed in healthcare, with an age range of 18 to 65 years. Two studies did not report an association; ten studies demonstrated a twofold increase in the chance of developing metabolic syndrome in shift workers as compared with day workers [25]. Five of the 12 studies were exclusively conducted in females. The authors suggest that the risk of metabolic syndrome seems to be higher in healthcare workers than in other industries. This might also be related to the high proportion of (mostly female) nurses examined in the respective studies, or to the irregular shifts in the healthcare sector.

Another systematic review investigated the association between shift work and metabolic syndrome, as well as obesity, dyslipidemia, hypertension and insulin resistance and concluded that treatment plans are needed for shift workers to manage disorders and other chronic diseases [23]. A meta-analytic study investigated the cross-sectional association between shift work and metabolic syndrome as well as the roles of sleep, sex and type of shift work in over 120,000 participants. The pooled odds ratio of metabolic syndrome in shift versus day workers was estimated as 1.14, thus much lower than that estimated by [25] and was no longer significant when cohort and case–control data were considered. Further, the odds ratio was significantly higher for those studies conducted only on females or males, compared to those in mixed samples, and rotating shift workers had stronger odds of metabolic syndrome than the other shift workers [12]. The higher prevalence of metabolic syndrome in nurses seems to hint once again at sex being one important factor within the association to shift work. However, another study in Korean female nurses [9] found a higher metabolic syndrome prevalence in non-shift working nurses than in shift working nurses. This is in line with our findings that female FORMER shift workers showed a lower BMI and higher HDL levels as compared to female NEVER shift workers. Further along this line, female PRESENT shift workers also presented the lowest trigyceride levels, compared to male shift workers, but also compared to female NEVER_ALL_ shift workers. Hence, our data suggest an interaction effect between sex and shift work group and it seems desirable for furture studies to model the differences and interactions between females and males more deeply. Specifically, female PRESENT shift workers might display a research group of interest. Jung et al. [9] argued that their observations might be related to a higher amount of physical activity of shift work nurses, as well as eating habits which can be related to a large proportion of variance in metabolic syndrome in nurses since caloric intake and specifically the number of calories eaten during evening hours explained more variance in metabolic syndrome risk than shift work. In this line, Vetter et al. [30] examined female nurses in a prospective study design, but with a particular focus on shift work schedule, chronotype and type 2 diabetes. There was only slight evidence that newly developed type 2 diabetes was higher in shift working nurses than day working nurses. Moreover, the relation to shift work was much more complex: the proportion of nurses with type 2 diabetes was not elevated in women working less than 10 years in shift work as compared with those working more than 10 years. Among early chronotypes, risk of type 2 diabetes was modestly reduced when working daytime schedules. In contrast, late chronotypes showed a significantly increased diabetes risk in day workers. Interestingly, this was attenuated if their work schedules included night shifts. These observations further hint at a mismatch between work schedule and chronotype which may explain some of the variance in developing type 2 diabetes and may also be considered for metabolic syndrome. In the present study, no sufficient data about the chronotype and the precise shift work schedules were available which, however, need as well to be addressed in future studies.

## Conclusion

In line with some previous studies, the present data do not support the hypothesis that night shift work can be considered a general risk factor for human health. This is at variance with other studies reporting hazardous effects of night shift work [2]. Potential explanations for these differences may be manifold and include inter alia individual differences in the adaptability to shift work [7], the socio-economic differences, particularly the quality of healthcare provision of study participants and the schedule of night shift work (fast rotating versus slowly rotating shifts). Also, the sleeping behavior and the chronotype may have an impact. All these data need to be recorded in future studies in order to clearly define the impact of night shift work on human health and to implement tailored health care programs to prevent significant effects of shift work on human health.

## Supplementary Information

Below is the link to the electronic supplementary material.

Supplementary file1 (DOCX 5208 KB)

## Data Availability

The datasets generated and/or analyzed during the current study are available from the Hein-Nixdorf-recall study comitee to other scientists on request in anonymized format and according to data protection policy in the ethics agreement. Data requests should be addressed to the corresponding author (n.bittner@fz-juelich.de).
