# Supplementary material for: Association between night shift work and markers of metabolism, cardiovascular and immune system in a population-based German cohort
Source: GeroScience. 2025 Mar 27;47(3):5141–55. doi: 10.1007/s11357-025-01596-8 (PMC12181476; doi:10.1007/s11357-025-01596-8)
Supplement: Supplementary file 1 — Supplementary file1 (DOCX 5208 KB) [file 11357_2025_1596_MOESM1_ESM.docx]

**Supplementary Material:**

**Supplementary Methods:**

***Supplementary information on sample size:***

We started our analyses with a total sample of 3087 + 2897 participants. Exclusion of participants due to missing data for each variable of interest is indicated in the flow chart in Supplementary Figure 1.

***Supplementary analysis***

As described in the main manuscript, we repeated our comparison between shift workers and MATCHED controls using all shift workers, i.e. regardless of medication (n= 125 for PRESENT and NEVER_PRES_; n = 662 for FORMER and NEVER_FORM_) in our sensitivity analyses. In all of these analyses number of medication was used as additional covariate.

However, we even considered further covariates, i.e. level of education and family status, in supplementary analyses.

Shift work, especially night shift work, has been repeatedly related to lower educational attainment compared to non-shift work, (Papantoniou et al., 2018; van Amelsvoort, Schouten, & Kok, 2004) and it has been discussed that lower educational attainment might be a selection criterion for shift work (Daghlas et al., 2021). Further, cardiometabolic risk factors show associations to education (Stephens et al., 2020), which is why we considered education as a confounding factor. In the current study, educational attainment was measured using the international standard classification of education (ISCED) (UNESCO, 1997) reflecting a higher education in a higher score.

Additionally, participants of the Heinz-Nixdorf-Recall Multigeneration study (MGS) comprise spouses, siblings, children or grand-children of Heinz-Nixdorf-recall (HNR) study participants. It might be assumed, that participants belonging to the same family share a quite similar socio-economic status as well as genetic vulnerability. Both have been linked to cardiometabolic status, socio-economic status as a determinant of metabolic health (Blanquet, Legrand, Pélissier, & Mourgues, 2019), while genome-wide association studies have linked several loci to the prevalence of metabolic syndrome or it´s components (Lind, 2019). To take both influencing factors into account, we considered the family status between both cohorts (MGS and HNR) as an additional confounding factor.

Thus, in our sensitivity analyses, all comparisons between matched samples (PRESENT vs NEVER_PRES_ shift workers; FORMER versus NEVER_FORM_) were repeated while number of medications was added as covariate. On top of that, education was added as a covariate and family status was added as a random effect with a constant term in our supplementary analyses. Lastly, all available participants of all three groups – PRESENT, FORMER and all NEVER shift workers (n = 3266) were again compared directly using an omnibus MANCOVA test correcting for number of medication (sensitivity analysis), as well as education and family status (supplementary analysis).

**Supplementary Results:**

**PRESENT versus matched NEVER_FORM_ shift workers:**

**Sensitivity analysis:**

There was no significant main effect of shift work as indicated by the MANCOVA (*p* = 0.13). In between subjects´ analyses, none of the mean values of the anthropometric and blood parameters differed significantly between PRESENT and NEVER_PRES_ workers except for number of erythrocytes (*p* = 0.011, Suppl. Table 1). PRESENT showed marginally (mean = 4.71 # / nl) lower values than NEVER_PRES_ shift workers (mean = 4.81 # / nl) with both groups presenting no pathological values.

Between subjects’ analyses indicated significant interactions between sex and shift work group for LDL-HDL-ratio (*p* = 0.047), levels of HDL (*p* = 0.008), similar to the main analysis, and CRP (*p* = 0.035). Here, the differences in HDL-levels and LDL-HDL-ratio between male and female participants were larger within PRESENT than in NEVER_PRES_ shift workers (Suppl. Figure 2). For levels of CRP, the differences in males and females were reversed in the two groups: For PRESENT shift workers, males showed lower values than females, in NEVER_PRES_ shift workers, males showed higher values than females. Thus, there were no effects of shift work on anthropometric and blood parameters per se. When introducing the number of relevant medications, as well as the different types of medication, as a covariate into the model, these results remained stable.

**Supplementary analysis:**

When correcting for education and family status in supplementary analyses (Suppl. Table 1), we found PRESENT shift workers (mean = 143.63) presenting higher triglyceride values than NEVER_PRES_ (mean = 109.50). Since this was not found when excluding participants with medication use, this is most likely driven by PRESENT shift workers with medication use. Further, PRESENT (mean = 4.71) showed marginally lower levels of erythrocytes than NEVER_PRES_ (mean = 4.81), which can be considered clinically irrelevant. The interaction effect between sex and shift work group for levels of LDL / HDL ratio (Suppl. Figure 2) was no longer significant (*p* = 0.065), similar for levels of CRP (*p* = 0.175) and LDL-HDL-ratio (*p* = 0.065) while we found an additional interaction effect for eosinophilic granulocytes [%] with PRESENT presenting marginally lower levels (mean = 2.81) than NEVER_PRES_ (mean = 3.02).

**FORMER versus matched NEVER_FORM_ shift workers:**

**Sensitivity analysis:**

There was no significant main effect of shift work group (FORMER versus NEVER_FORM_) on anthropometric and blood parameters as indicated by the MANCOVA (*p* = 0.190). Between subjects’ analyses indicated marginal differences in mean values below the clinically relevant cut-offs for waist-hip-ratio (*p* = 0.028; FORMER mean = 0.94, NEVER_FORM_ mean = 0.95) and levels of HDL (*p* = 0.030, FORMER mean = 56.27; NEVER_FORM_ mean = 58.02, Suppl. Table 2, Suppl. Figure 3) but not in any other parameter.

Between subjects’ effects indicated a significant interaction effect between shift group and sex only for HbA1c (*p* = 0.046, Suppl. Figure 3), since female FORMER shift workers showed lower values than all other groups (female and male NEVER_FORM_ and male FORMER). We did not find a significant interaction effect for BMI, for which we had found a significant interaction effect in our main analyses. When introducing the number of relevant medications, as well as the different types of medication, as a covariate into the model these results remained stable.

**Supplementary analysis:**

When correcting for education and family status, we obtained a very similar result pattern, with marginally higher mean waist-to-hip-ratios (FORMER = 0.95; NEVER_FORM_ = 0.94) and marginally lower mean HDL-values for FORMER shift workers (56.27) compared to NEVER_FORM_ (58.02), which did not present values higher than the clinical cutoff (Suppl. Table 2).

**Analyses using all available participants:**

When comparing all three groups – PRESENT, FORMER and all NEVER shift workers (n = 3266) - directly using an omnibus MANCOVA test, correcting for education as covariate and family status as a random effect, the above-mentioned results stayed largely the same. However, there was a statistically significant difference in waist-hip-ratio (whr) between shift work groups. But looking at the mean differences, these effects seem to reflect clinically or biologically non-meaningful differences (mean whr: PRESENT = 0.89; FORMER = 0.95; NEVER = 0.90). The same was also true for numbers of leukocytes and erythrocytes (Suppl. Table 3). However, the interaction effect between sex and shift work group on LDL / HDL ratio was again significant (*p* = 0.023) with female PRESENT shift workers showing the lowest ratio (residual means of males: PRESENT = 2.72, FORMER = 2.61, NEVER_all_ = 2.49; residual means of females: PRESENT = 1.76, FORMER = 2.08; NEVER = 2.01, please compare to Suppl. Figure 2). Further, this analysis showed that the higher triglyceride levels in PRESENT than NEVER_PRES_ shift workers (when analyzing participants with and without medication, Suppl. Table 1) may be driven by an interaction with sex, since the group difference was no longer significant. Rather, male PRESENT shift workers showed the highest values, but female PRESENT shift workers the lowest values (Suppl. Figure 4).

|  | **Mean values** | | ***p*-value** | | ***p*-value corrected for education & family status** | |
| --- | --- | --- | --- | --- | --- | --- |
| **Parameter** | **PRESENT** | **NEVER_PRES_** | **Group differences** | **Interaction term** | **Group differences** | **Interaction term** |
| Systolic BP [mmHg] | 125.34 | 124.00 | 0.721 | 0.512 | 0.928 | 0.355 |
| Diastolic BP [mmHg] | 75.67 | 76.06 | 0.896 | 0.306 | 0.621 | 0.159 |
| LDL / HDL ratio | 2.38 | 2.28 | 0.671 | 0.047* | 0.664 | 0.065 |
| Body Mass Index [kg/m^2^ | 27.06 | 26.63 | 0.499 | 0.985 | 0.713 | 0.568 |
| Waist-Hip-Ratio | 0.89 | 0.89 | 0.911 | 0.216 | 0.774 | 0.129 |
| HDL-Cholesterol [mg/dl] | 57.41 | 58.29 | 0.885 | 0.008* | 0.884 | 0.014* |
| LDL-cholesterol [mg/dl] | 126.54 | 124.94 | 0.746 | 0.819 | 0.761 | 0.979 |
| Total cholesterol [mg/dl] | 204.33 | 199.91 | 0.344 | 0.859 | 0.335 | 0.559 |
| Triglycerides [mg/dl] | 143.63 | 109.50 | 0.068 | 0.065 | 0.035* | 0.054 |
| HbA1C [%] | 5.56 | 5.55 | 0.991 | 0.579 | 0.390 | 0.839 |
| Glucose (serum) [mg/dl] | 97.59 | 95.90 | 0.581 | 0.766 | 0.759 | 0.711 |
| Uric acid [mg/dl] | 5.52 | 5.59 | 0.571 | 0.758 | 0.31 | 0.754 |
| C-reactive protein [mg/dl] | .20 | .20 | 0.987 | 0.035* | 0.516 | 0.175 |
| Leukocytes [# / nl] | 6.03 | 5.96 | 0.011* | 0.325 | 0.673 | 0.945 |
| Erythrocytes [# / nl] | 4.71 | 4.81 | 0.862 | 0.732 | 0.012* | 0.432 |
| Basophilic granulocytes [%] | .52 | .54 | 0.947 | 0.097 | 0.966 | 0.148 |
| Eosinophilic granulocytes[%] | 2.81 | 3.02 | 0.694 | 0.053 | 0.615 | 0.044* |
| Neutrophilic granulocytes[%] | 56.90 | 57.001 | 0.768 | 0.440 | 0.747 | 0.451 |

**Supplementary Table 1.** *Mean values for each parameter, as well as p-values for the group differences between PRESENT and matched NEVER_PRES_ shift workers, as well as the interaction term between sex and shift work group as obtained with MANCOVA with Bonferroni correction for multiple comparisons, * = p < 0.05.*

|  | **Mean values** | | **p-value** | | ***p*-value corrected for education & family status** | |
| --- | --- | --- | --- | --- | --- | --- |
| **Parameter** | **FORMER** | **NEVER_FORM_** | **Group differences** | **Interaction term** | **Group differences** | **Interaction term** |
| Systolic BP [mmHg] | 128.54 | 128.76 | 0.712 | 0.729 | 0.623 | 0.854 |
| Diastolic BP [mmHg] | 75.51 | 76.12 | 0.132 | 0.310 | 0.094 | 0.447 |
| LDL / HDL ratio | 2.48 | 2.35 | 0.072 | 0.705 | 0.086 | 0.629 |
| Body Mass Index [kg/m^2^] | 28.19 | 27.69 | 0.399 | 0.086 | 0.407 | 0.189 |
| Waist-Hip-Ratio | 0.95 | 0.94 | 0.028* | 0.172 | 0.04 | 0.083 |
| HDL-Cholesterol [mg/dl] | 56.27 | 58.02 | 0.030* | 0.468 | 0.033 | 0.315 |
| LDL-cholesterol [mg/dl] | 126.11 | 127.01 | 0.893 | 0.273 | 0.809 | 0.248 |
| Total cholesterol [mg/dl] | 205.13 | 206.60 | 0.893 | 0.346 | 0.921 | 0.382 |
| Triglycerides [mg/dl] | 135.86 | 127.61 | 0.075 | 0.679 | 0.111 | 0.553 |
| HbA1C [%] | 5.83 | 5.82 | 0.507 | 0.046* | 0.458 | 0.066 |
| Glucose (serum) [mg/dl] | 102.52 | 101.33 | 0.739 | 0.375 | 0.643 | 0.647 |
| Uric acid [mg/dl] | 6.09 | 6.01 | 0.512 | 0.465 | 0.591 | 0.559 |
| C-reactive protein [mg/dl] | 0.28 | 0.23 | 0.192 | 0.505 | 0.22 | 0.576 |
| Leukocytes [# / nl] | 6.41 | 6.14 | 0.668 | 0.224 | 0.385 | 0.461 |
| Erythrocytes  [# / nl] | 4.74 | 4.77 | 0.294 | 0.302 | 0.925 | 0.085 |
| Basophilic granulocytes [%] | 0.51 | 0.50 | 0.444 | 0.840 | 0.39 | 0.796 |
| Eosinophilic granulocytes [%] | 2.80 | 2.95 | 0.390 | 0.549 | 0.297 | 0.693 |
| Neutrophilic granulocytes [%] | 59.44 | 58.87 | 0.648 | 0.289 | 0.746 | 0.468 |

**Supplementary Table 2.** *Mean values for each parameter, as well as p-values for the group differences between FORMER and matched NEVER_FORM_ shift workers, as well as the interaction term between sex and shift work group as obtained with MANCOVA with Bonferroni correction for multiple comparisons, * = p < 0.05.*

|  | **Mean values** | | | ***p*-value** | |
| --- | --- | --- | --- | --- | --- |
| Parameter | **PRESENT** | **FORMER** | **NEVER_ALL_** | **Group differences** | **Interaction term** |
| Systolic BP [mmHg] | 125.34 | 128.54 | 126.78 | 0.307 | 0.761 |
| Diastolic BP [mmHg] | 75.67 | 75.51 | 74.97 | 0.144 | 0.866 |
| LDL / HDL ratio | 2.38 | 2.48 | 2.21 | 0.078 | 0.023* |
| Body Mass Index [kg/m^2^ | 27.06 | 28.19 | 27.42 | 0.196 | 0.553 |
| Waist-Hip-Ratio | 0.89 | 0.95 | 0.90 | 0.049* | 0.477 |
| HDL-Cholesterol [mg/dl] | 57.41 | 56.27 | 62.48 | 0.105 | 0.098 |
| LDL-cholesterol [mg/dl] | 126.54 | 126.11 | 128.80 | 0.651 | 0.187 |
| Total cholesterol [mg/dl] | 204.33 | 205.13 | 212.90 | 0.192 | 0.076 |
| Triglycerides [mg/dl] | 143.63 | 135.86 | 122.84 | 0.078 | < 0.001* |
| HbA1C [%] | 5.56 | 5.83 | 5.79 | 0.704 | 0.240 |
| Glucose (serum) [mg/dl] | 97.59 | 102.52 | 99.37 | 0.983 | 0.933 |
| Uric acid [mg/dl] | 5.52 | 6.09 | 5.60 | 0.456 | 0.488 |
| C-reactive protein [mg/dl] | 0.20 | 0.28 | 0.24 | 0.067 | 0.334 |
| Leukocytes [# / nl] | 6.03 | 6.41 | 6.05 | 0.018* | 0.699 |
| Erythrocytes [# / nl] | 4.71 | 4.74 | 4.66 | 0.012* | 0.031* |
| Basophilic granulocytes [%] | 0.52 | 0.51 | 0.53 | 0.483 | 0.234 |
| Eosinophilic granulocytes [%] | 2.81 | 2.80 | 2.74 | 0.876 | 0.743 |
| Neutrophilic granulocytes [%] | 56.90 | 59.44 | 59.16 | 0.394 | 0.869 |

***Supplementary Table 3.*** *Shows mean values for each group for each parameter. Presented are p-values for the group differences between PRESENT, FORMER and all available NEVER_ALL_ shift workers, as well as the interaction term between sex and shift work group as obtained with MANCOVA with Bonferroni correction for multiple comparisons* *corrected for education and family status, * = p < 0.05. Please note that mean values for PRESENT and FORMER shift workers are the same as for suppl. Table 2 & 3 and are presented for comparison to NEVER shift workers only.*


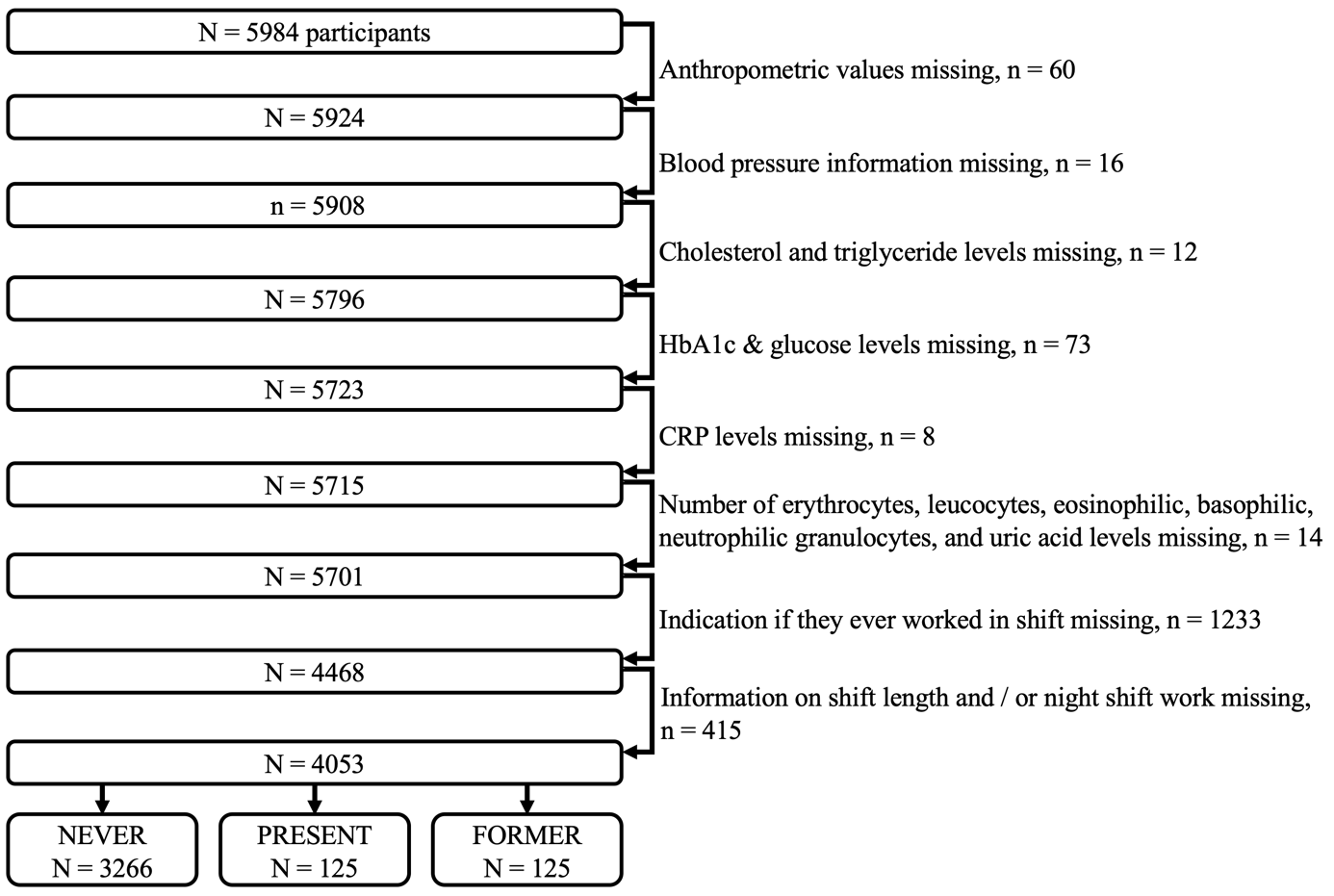


***Supplementary Figure 1****: Flow chart indicating missing values for each variable of interest.*

PRESENT

NEVER_PRES_

m

f

m

f

PRESENT

NEVER_PRES_

m

f

m

f

PRESENT

NEVER_PRES_

m

f

m

f

***Supplementary Figure 2****: Residual means (corrected for age) for the significant interaction between shift work group (PRESENT and* NEVER_PRES_*) and sex for LDL-HDL ratio, levels of HDL and CRP. Error bars represent standard errors of the mean.*

FORMER

NEVER_FORM_

m

f

m

f

FORMER

NEVER_FORM_

m

f

m

f

FORMER

NEVER_FORM_

m

f

m

f

***Supplementary Figure 3****: Residual means (corrected for age) for the significant main effect between FORMER and NEVER_FORM_ shift workers in levels of HDL and Waist-Hip-Ratio, as well as for the significant interaction between sex and shift work group and levels of HbA1c. Error bars represent standard errors of the mean.*

***Supplementary Figure 4****: Residual means (corrected for age, education and family status) for the significant interaction between sex and shift work group and levels of triglycerides. Error bars represent standard errors of the mean.*
